# A combined prediction model for biliary tract cancer using the prognostic nutritional index and pathological findings: a single-center retrospective study

**DOI:** 10.1186/s12876-021-01957-5

**Published:** 2021-10-13

**Authors:** Masashi Utsumi, Koji Kitada, Naoyuki Tokunaga, Takamitsu Kato, Toru Narusaka, Ryosuke Hamano, Hideaki Miyasou, Yousuke Tsunemitsu, Shinya Otsuka, Masaru Inagaki

**Affiliations:** Department of Surgery, National Hospital Organization Fukuyama Medical Center, 4-14-17 Okinogami-cho, Fukuyama City, Hiroshima 720-8520 Japan

**Keywords:** Biliary tract cancer, Inflammation-based prognostic score, Prognosis, Prognostic nutritional index, Surgical resection

## Abstract

**Background:**

The prognostic nutritional index, a marker of nutritional status and systemic inflammation, is a known biomarker for various cancers. However, few studies have evaluated the predictive value of the prognostic nutritional index in patients with biliary tract cancer. Therefore, we investigated the prognostic significance of the prognostic nutritional index, and developed a risk-stratification system to identify prognostic factors in patients with biliary tract cancer.

**Methods:**

Between July 2010 and March 2021, 117 patients with biliary tract cancer were recruited to this single-center, retrospective study. The relationship between clinicopathological variables, including the prognostic nutritional index, and overall survival was analyzed using univariate and multivariate analyses. A *P* < 0.05 was considered statistically significant.

**Results:**

The median age was 75 (range 38–92) years. Thirty patients had intrahepatic cholangiocarcinoma; 29, gallbladder carcinoma; 27, distal cholangiocarcinoma; 17, ampullary carcinoma; and 13, perihilar cholangiocarcinoma. Curative (R0) resection was achieved in 99 patients. In univariate analysis, the prognostic nutritional index (< 42), lymph node metastasis, carbohydrate antigen 19-9 level (> 20 U/mL), preoperative cholangitis, tumor differentiation, operation time (≥ 360 min), and R1–2 resection were significant risk factors for overall survival. The prognostic nutritional index (*P* = 0.027), lymph node metastasis (*P* = 0.040), and tumor differentiation (*P* = 0.006) were independent prognostic factors in multivariate analysis. A combined score of the prognostic nutritional index and pathological findings outperformed each marker alone, in terms of discriminatory power.

**Conclusions:**

The prognostic nutritional index, lymph node metastasis, and tumor differentiation were independent prognostic factors after surgical resection in patients with biliary tract cancer. A combined prediction model using the prognostic nutritional index and pathological findings accurately predicted prognosis, and can be used as a novel prognostic factor in patients with biliary tract cancer.

## Background

Biliary tract cancer (BTC), including gallbladder carcinoma (GBC), cholangiocarcinoma, and ampullary carcinoma, is a relatively rare, but aggressive malignancy [1]. Despite its rarity, the incidence of BTC has steadily increased in recent decades [1]. Radical resection is the only curative treatment option for BTC. However, the high recurrence rate is a major concern [2]. Moreover, BTC is usually diagnosed at an advanced stage, at which point most patients cannot be considered as candidates for radical resection. Despite recent developments in surgical techniques and adjuvant chemotherapy, the prognosis remains poor [3, 4]. Preoperative prognostic factors for BTC may allow better risk–benefit assessment before surgery, and permit patient stratification for more personalized treatment [5]. Therefore, it is essential to identify new predictive biomarkers.

The prognostic nutritional index (PNI) is a marker of nutritional status and systemic inflammation, based on serum albumin concentration and total lymphocyte count, both of which can be easily obtained from routine preoperative blood tests [6]. Several studies [7–11] have shown that the PNI can be used as a prognostic marker in patients with various cancers. However, few studies have evaluated the prognostic value of the PNI in patients with BTC. Therefore, in this single-center, retrospective study, we investigated the prognostic significance of the PNI in patients with BTC, and explored its potential clinical application. We also compared the PNI to other inflammation-based prognostic scores, including the Glasgow Prognostic Score (GPS) [12], C-reactive protein (CRP) to albumin ratio (CAR) [13], neutrophil to lymphocyte ratio (NLR), and platelet to lymphocyte ratio (PLR) [14, 15]. Furthermore, we developed a risk-stratification system combining clinicopathological predictors to identify prognostic factors in patients with BTC.

## Methods

### Patients

A total of 117 consecutive patients who underwent surgical resection for BTC at the Department of Surgery, National Hospital Organization Fukuyama Medical Center, Hiroshima, Japan, between July 2010 and March 2021 were retrospectively reviewed. BTC included GBC, intrahepatic cholangiocarcinoma (ICC), distal cholangiocarcinoma, ampullary carcinoma, and perihilar cholangiocarcinoma, as confirmed by imaging and pathological examination. One patient died of heart failure due to arrhythmia on postoperative day 18 during the perioperative period. This patient was excluded from the study. Consequently, a total of 116 patients who underwent surgical resection for BTC were analyzed.

### Data collection

Clinicopathological data were obtained retrospectively from patients’ medical records, including demographic information (age at surgery and sex), laboratory data (CRP level, serum albumin concentration, platelet count, neutrophil count, lymphocyte count, and tumor markers), comorbidities (hypertension, diabetes mellitus, cardiac disease, and stroke), preoperative cholangitis, operative procedure (i.e., type of resection), operative blood loss, operation time, transfusion, tumor stage (Union for International Cancer Control Tumor–Node–Metastasis [TNM] classification [sixth edition]) [16], tumor differentiation, and postoperative adjuvant chemotherapy. The extent of hepatic resection was classified according to the Brisbane 2000 nomenclature [17]. Major hepatectomy was defined as the resection of ≥ 3 contiguous liver segments, according to Couinaud’s classification [18], while minor hepatectomy was defined as the resection of < 3 contiguous liver segments, or nonanatomic partial resection. Curative (R0) resection was defined as complete removal of all macroscopic nodules with microscopically clear margins. R1 and R2 resections were defined as microscopic or macroscopic disease, respectively, involving ≥ 1 margin. Complications were defined according to the Clavien–Dindo classification [19]. In this study, postoperative complications were defined as complications of Clavien–Dindo grade ≥ IIIa. Postoperative mortality was defined as death from any cause within 30 days after surgery.

### PNI and other inflammation-based prognostic scores

Peripheral venous blood samples were collected within 2 weeks before surgery. The PNI was calculated as 10 × serum albumin concentration (g/dL) + 0.05 × total lymphocyte count (/mm^3^) [6, 20]. The GPS was defined as follows: a normal serum albumin concentration (≥ 3.5 g/dL) and CRP level (≤ 1.0 mg/dL) was scored as 0, a low serum albumin concentration (< 3.5 g/dL) or a high CRP level (> 1.0 mg/dL) was scored as 1, and a low serum albumin concentration (< 3.5 g/dL) and a high CRP level (> 1.0 mg/dL) was scored as 2 [12]. The CAR was calculated by dividing the serum CRP level (mg/dL) by the serum albumin concentration (g/dL) [13]. The NLR and PLR were calculated by dividing the neutrophil or platelet count, respectively, by the lymphocyte count [14, 15].

### Follow-up

All patients underwent routine follow-up until March 2021. Postoperative follow-up included medical history (symptoms and physical examination), laboratory tests, and imaging studies performed every 6 months for ≥ 5 years. Patients with lymph node metastasis or who underwent R1–2 resection received postoperative adjuvant chemotherapy (tegafur/gimeracil/oteracil) for approximately 6 months.

### Outcomes

The relationship between clinicopathological variables, including the PNI, and overall survival (OS) was analyzed using univariate and multivariate analyses. OS was defined as the interval between surgery and death or last follow-up. Disease-free survival (DFS) was defined as the interval between surgery and recurrence. A combined prediction model was developed using independent prognostic factors. The area under the receiver operating characteristic curve (AUC) was calculated to compare the predictive ability of each scoring system.

### Statistical analyses

Data are expressed as the mean ± standard deviation. Univariate analysis was performed using the Mann–Whitney *U* test and Chi-square test. Diagnostic accuracy was determined using the AUC method. The optimal cutoff values of the PNI and other inflammation-based prognostic scores were determined by maximizing the Youden index (sensitivity + specificity − 1) [21]. OS and DFS rates were estimated using the Kaplan–Meier method, and compared using the log-rank test. Univariate and multivariate analyses were performed using the Cox proportional hazards model. Prognostic factors that were statistically significant in the univariate analysis were included in the multivariate analysis. All statistical analyses were conducted using JMP (version 11; SAS Institute, Cary, NC, USA). Statistical significance was defined as *P* < 0.05.

## Results

### Patient characteristics

Patient characteristics are summarized in Table 1. The median age was 75 (range 38–92) years. Thirty patients had ICC; 29, GBC; 27, distal cholangiocarcinoma; 17, ampullary carcinoma; and 13, perihilar cholangiocarcinoma. Curative (R0) resection was achieved in 99 patients. Operative procedures included pancreaticoduodenectomy in 43 patients, major hepatectomy in 35, minor hepatectomy in 25, cholecystectomy in 11, hepatopancreaticoduodenectomy in two, and bile duct resection without hepatectomy in one. None of the patients received neoadjuvant chemotherapy or underwent preoperative portal vein embolization. Postoperative complications were observed in 44 of 116 patients: pancreatic fistula in 23 patients, bile leakage in eight, abdominal abscess in eight, pleural effusion in two, abdominal bleeding in one, and chylous ascites in one. The postoperative mortality rate was 0.0%. The optimal cutoff value of the PNI was 42. Patients were stratified into a high PNI (≥ 42) group (n = 88; 75.9%) and a low PNI (< 42) group (n = 28; 24.1%), according to the cutoff value.

**Table 1:** Patient characteristics

Characteristic	Patients
Age (years), mean ± SD (range)	74.0 ± 9.55 (39–92)
Sex (male/female)	73/43
BMI (kg/m^2^), mean ± SD (range)	22.39 ± 3.65 (14.20–32.46)
Preoperative laboratory data, mean ± SD (range)	
Albumin concentration (g/dL)	3.79 ± 0.53 (1.70–4.80)
Platelet count (× 10^4^/mm^3^)	21.65 ± 66.61 (3.37–46.30)
Neutrophil count (× 10^3^/mm^3^)	3.75 ± 1.88 (1.01–15.39)
Lymphocyte count (× 10^3^/mm^3^)	1.58 ± 0.74 (0.48–5.80)
CRP level (mg/dL)	1.16 ± 2.77 (0.01–24.18)
CEA level (ng/mL)	6.02 ± 13.12 (0.56–113.06)
CA19-9 level (U/mL)	1 019.91 ± 4 156.75 (2.00–39,284.20)
PNI	45.79 ± 6.53 (22.21–62.98)
GPS (0/1/2)	72/33/11
CAR	0.39 ± 1.41 (0.002–14.22)
NLR	2.80 ± 2.24 (0.75–14.76)
PLR	158 ± 85 (41–561)
Type of cancer, n (%)	
Intrahepatic cholangiocarcinoma	30 (25.9)
Gallbladder carcinoma	29 (25.0)
Distal cholangiocarcinoma	27 (23.3)
Ampullary carcinoma	17 (14.7)
Perihilar cholangiocarcinoma	13 (11.2)
Preoperative cholangitis, n (%)	43 (37.1)
Comorbidities (absent/present)	34/82
Surgical procedure, n (%)	
Cholecystectomy	11 (9.5)
Bile duct resection w/o liver resection	1 (0.9)
Minor hepatectomy	25 (21.6)
Major hepatectomy	35 (30.2)
Pancreaticoduodenectomy	42 (36.2)
Hepatopancreaticoduodenectomy	2 (1.7)
Operation time (minutes), mean ± SD (range)	457.1 ± 171.1 (124–1049)
Blood loss (mL), mean ± SD (range)	772.2 ± 1 507.8 (10–13,870)
Blood transfusion, n (%)	16 (13.8)
T stage (1/2/3/4)	18/39/51/8
N stage (1), n (%)	47 (40.5)
UICC stage (sixth edition) (0/I/II/III/IV)	3/17/48/31/16
Resection (R0/R1/R2)	98/15/3
Tumor differentiation (well/moderate/poor/pap/well-pap/other/unknown)	43/32/9/7/5/7/13
Postoperative adjuvant chemotherapy, n (%)	80 (69.0)
Postoperative complications (Clavien–Dindo grade ≥ IIIa) (absent/present)	43/73

*BMI* body mass index, *CA19-9* carbohydrate antigen 19-9, *CAR* CRP to albumin ratio, *CEA* carcinoembryonic antigen, *CRP* C-reactive protein, *GPS* Glasgow Prognostic Score, *NLR* neutrophil to lymphocyte ratio, *PLR* platelet to lymphocyte ratio, *PNI* prognostic nutritional index, *SD* standard deviation, *UICC* Union for International Cancer Control, *w/o* without

### Relationship between clinicopathological variables and the PNI

Table 2 shows the relationship between clinicopathological variables and the PNI. Patients in the low PNI (< 42) group had a significantly longer mean operation time than those in the high PNI (≥ 42) group (514 ± 220 *vs.* 438 ± 149 min, respectively; *P* = 0.043). A significantly higher proportion of patients had lymph node metastasis in the low PNI (< 42) group than in the high PNI (≥ 42) group.

**Table 2:** Clinicopathological characteristics according to the PNI

Characteristic	High PNI (≥ 42) (n = 88)	Low PNI (< 42) (n = 28)	*P*-value
Age (years), mean ± SD	73.3 ± 9.2	75.6 ± 10.9	0.271
Sex (male/female)	54/34	19/9	0.533
BMI (kg/m^2^), mean ± SD	22.6 ± 3.4	21.8 ± 4.4	0.336
CEA level (ng/mL), mean ± SD	6.35 ± 14.90	4.97 ± 4.26	0.631
CA19-9 level (U/mL), mean ± SD	1 072.7 ± 4 565.5	852.8 ± 2 528.4	0.809
Preoperative cholangitis (absent/present)	58/30	15/13	0.243
Comorbidities (absent/present)	25/63	9/19	0.707
Type of cancer (ICC/other)	23/65	7/21	0.905
Primary disease			0.130
ICC	23	7	
GBC	22	7	
Distal cholangiocarcinoma	20	7	
Ampullary carcinoma	16	1	
Perihilar cholangiocarcinoma	7	6	
Surgical procedure			
Cholecystectomy	9	2	0.311
Bile duct resection w/o liver resection	1	0	
Minor hepatectomy	21	4	
Major hepatectomy	22	13	
Pancreaticoduodenectomy	34	8	
Hepatopancreaticoduodenectomy	1	1	
Resection (R0–1/R2)	77/11	21/7	0.306
Operation time (minutes), mean ± SD	438 ± 149	514 ± 220	0.043*
Blood loss (mL), mean ± SD	717 ± 1 560	943 ± 1 341	0.491
Transfusion (no/yes)	79/9	21/7	0.062
T stage (≥ 3), n (%)	43 (48.9)	16 (57.1)	0.445
N stage (1), n (%)	30 (34.1)	17 (60.7)	0.013*
UICC stage (sixth edition) (I–II/III–IV)	56/32	12/16	0.053
Tumor differentiation (well/other)	37/51	11/17	0.796
Postoperative complications (Clavien–Dindo grade ≥ IIIa) (absent/present)	56/32	17/11	0.781
Postoperative adjuvant chemotherapy (no/yes)	28/60	8/20	0.745

*BMI* body mass index, *CA19-9* carbohydrate antigen 19-9, *CEA* carcinoembryonic antigen, *GBC* gallbladder carcinoma, *ICC* intrahepatic cholangiocarcinoma, *PNI* prognostic nutritional index, *SD* standard deviation, *UICC* Union for International Cancer Control, *w/o* without

^*^P < 0.05; **P < 0.01

### Univariate and multivariate analyses of clinicopathological factors affecting OS after surgical resection

**Fig. 1:**
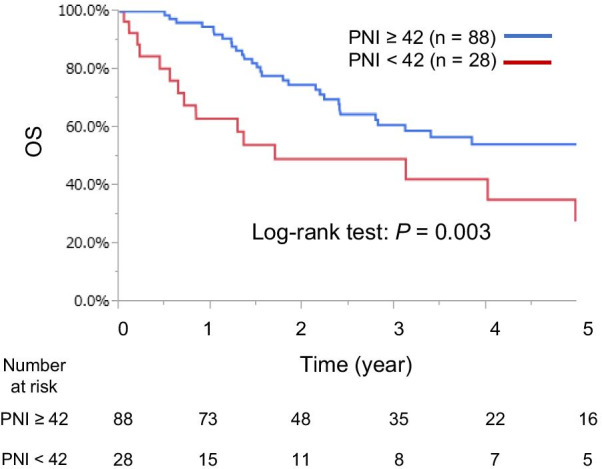
Kaplan–Meier curves of OS after resection of BTC in patients stratified by the PNI. *BTC* biliary tract cancer, *OS* overall survival, *PNI* prognostic nutritional index

The median OS was 43.9 (range 1–119.7) months. The 1-, 3-, and 5-year OS rates were 85.6%, 60.7%, and 34.6%, respectively. In the Kaplan–Meier analysis of all patients, the low PNI (< 42) group had a significantly shorter OS than the high PNI (≥ 42) group (*P* = 0.003; Fig. 1). Table 3 shows the relationship between clinicopathological variables, including the PNI, and OS after surgical resection. In univariate analysis, OS was significantly worse in patients with lymph node metastasis (*P* < 0.001), T stage ≥ 3 (*P* < 0.001), carbohydrate antigen 19-9 levels ≥ 20 U/mL (*P* = 0.013), a low PLR (< 119) (*P* = 0.003), preoperative cholangitis (*P* = 0.049), tumor differentiation (*P* = 0.003), an operation time ≥ 360 min (*P* = 0.032), and R1–2 resection (*P* < 0.001). Multivariate analysis showed that a low PNI (< 42) (*P* = 0.027), lymph node metastasis (*P* = 0.040), and tumor differentiation (*P* = 0.006) were significant independent predictors of OS.

**Table 3:** Univariate and multivariate analyses of clinicopathological factors affecting OS after resection of BTC

Factor	Univariate analysis		Multivariate analysis	
	n	*P*-value	HR (95% CI)	*P*-value
Age (years)		0.943	–	–
≥ 75	60			
< 75	56			
Sex		0.090	–	–
Male	73			
Female	43			
BMI (kg/m^2^)		0.460	–	–
≥ 20	80			
< 20	36			
CEA level (ng/mL)		0.847	–	–
≥ 9	17			
< 9	99			
CA19-9 level (U/mL)		0.013*	1.12 (0.57–2.23)	0.733
≥ 20	56			
< 20	60			
Preoperative cholangitis		0.049*	1.14 (0.62–2.08)	0.653
Present	43			
Absent	73			
Comorbidities		0.095	–	–
Present	82			
Absent	34			
Primary disease		0.090	–	–
ICC	30			
GBC	29			
Distal cholangiocarcinoma	27			
Ampullary carcinoma	17			
Perihilar cholangiocarcinoma	13			
Surgical procedure		0.540	–	–
Cholecystectomy	11			
Bile duct resection w/o liver resection	1			
Minor hepatectomy	25			
Major hepatectomy	35			
Pancreaticoduodenectomy	42			
Hepatopancreaticoduodenectomy	2			
Resection		< 0.001***	1.26 (0.59–2.59)	0.536
R0	98			
R1–2	18			
Operation time (minutes)		0.032 *	1.03 (0.49–2.28)	0.948
≥ 360	82			
< 360	34			
Blood loss (mL)		0.550	–	–
≥ 200	81			
< 200	35			
Transfusion		0.612	–	–
No	100			
Yes	16			
T stage		< 0.001***	1.92 (0.97–4.08)	0.075
< 3	57			
≥ 3	59			
N stage		< 0.001***	1.99 (1.03–3.90)	0.040*
0	69			
1	47			
Tumor differentiation		0.003**	2.45 (1.29–4.83)	0.006**
Well	48			
Other	68			
PNI		0.003**	2.12 (1.09–4.04)	0.027**
< 42	28			
≥ 42	88			
GPS		0.065	–	–
0	44			
1–2	72			
CAR		0.064	–	–
< 1	65			
≥ 1	51			
NLR		0.352	–	–
< 2.55	85			
≥ 2.55	31			
PLR		0.014*	1.39 (0.69–2.92)	0.357
< 119	68			
≥ 119	48			
Postoperative complications (Clavien–Dindo grade ≥ IIIa)		0.594	–	–
Absent	73			
Present	43			
Postoperative adjuvant chemotherapy		0.518	–	–
No	36			
Yes	80			

*BMI* body mass index, *BTC* biliary tract cancer, *CA19-9* carbohydrate antigen 19-9, *CAR* CRP to albumin ratio, *CEA* carcinoembryonic antigen, *CI* confidence interval, *CRP* C-reactive protein, *GBC* gallbladder carcinoma, *GPS* Glasgow Prognostic Score, *HR* hazard ratio, *ICC* intrahepatic cholangiocarcinoma, *NLR* neutrophil to lymphocyte ratio, *OS* overall survival, *PLR* platelet to lymphocyte ratio, *PNI* prognostic nutritional index, *w/o* without

^*^P < 0.05; **P < 0.01; ***P < 0.001

### Univariate and multivariate analyses of clinicopathological factors affecting DFS after surgical resection

The median DFS was 27.3 (range 1–104) months. The 1-, 3-, and 5-year DFS rates were 57.7%, 44.2%, and 37.5%, respectively. Table 4 shows the relationship between clinicopathological variables, including the PNI, and DFS after surgical resection. In univariate analysis, DFS was significantly worse in patients with lymph node metastasis (*P* < 0.001), T stage ≥ 3 (*P* < 0.001), a low PNI (< 42) (*P* = 0.008), a high CAR (≥ 1) (*P* = 0.012), carbohydrate antigen 19-9 levels ≥ 20 U/mL (*P* = 0.009), preoperative cholangitis (*P* = 0.027), tumor differentiation (*P* = 0.001), an operation time ≥ 360 min (*P* = 0.006), and R1–2 resection (*P* < 0.001). Multivariate analysis showed that tumor differentiation (*P* = 0.016) was a significant independent predictor of DFS.

**Table 4:** Univariate and multivariate analyses of clinicopathological factors affecting DFS after resection of BTC

Factor	Univariate analysis		Multivariate analysis	
	n	*P*-value	HR (95% CI)	*P*-value
Age (years)		0.316	–	–
≥ 75	60			
< 75	56			
Sex		0.078	–	–
Male	73			
Female	43			
BMI (kg/m^2^)		0.294	–	–
≥ 20	80			
< 20	36			
CEA level (ng/mL)		0.413	–	–
≥ 9	17			
< 9	99			
CA19-9 level (U/mL)			1.21 (0.63–2.47)	0.556
≥ 20	56	0.009**		
< 20	60			
Preoperative cholangitis		0.027*	1.18 (0.57–2.40)	0.644
Present	43			
Absent	73			
Comorbidities		0.184	–	–
Present	82			
Absent	34			
Primary disease		0.060	–	–
ICC	30			
GBC	29			
Distal cholangiocarcinoma	27			
Ampullary carcinoma	17			
Perihilar cholangiocarcinoma	13			
Surgical procedure		0.115	–	–
Cholecystectomy	11			
Bile duct resection w/o liver resection	1			
Minor hepatectomy	25			
Major hepatectomy	35			
Pancreaticoduodenectomy	42			
Hepatopancreaticoduodenectomy	2			
Resection		< 0.001***	2.35 (0.44–43.51)	
R0	98			0.604
R1–2	18			
Operation time (min)		0.006**	1.07 (0.521–2.20)	0.851
≥ 360	82			
< 360	34			
Blood loss (mL)		0.478	–	–
≥ 200	81			
< 200	35			
Transfusion			–	–
No	100	0.293		
Yes	16			
T stage		< 0.001***	1.79 (0.78–4.17)	0.171
< 3	57			
≥ 3	59			
N stage		< 0.001***	1.30 (0.62–2.89)	0.449
0	69			
1	47			
Tumor differentiation		0.001**	2.19 (1.16–4.24)	0.016*
Well	48			
Other	68			
PNI		0.008**	1.29 (0.58–3.08)	0.540
< 42	28			
≥ 42	88			
GPS			–	–
0	44	0.126		
1–2	72			
CAR		0.012*	1.29 (0.64–2.50)	0.469
< 1	65			
≥ 1	51			
NLR		0.500	–	–
< 2.55	85			
≥ 2.55	31			
PLR		0.269	–	–
< 119	68			
≥ 119	48			
Postoperative complications (Clavien–Dindo grade ≥ IIIa)		0.449	–	–
Absent	73			
Present	43			
Postoperative adjuvant chemotherapy		0.222	–	–
No	36			
Yes	80			

*BMI* body mass index, *BTC* biliary tract cancer, *CA19-9* carbohydrate antigen 19-9, *CEA* carcinoembryonic antigen, *CI* confidence interval, *CAR* CRP to albumin ratio, *CRP* C-reactive protein, *GBC* gallbladder carcinoma, *GPS* Glasgow Prognostic Score, *HR* hazard ratio, *ICC* intrahepatic cholangiocarcinoma, *NLR* neutrophil to lymphocyte ratio, *OS* overall survival, *PLR* platelet to lymphocyte ratio, *PNI* prognostic nutritional index

^*^P < 0.05; **P < 0.01; ***P < 0.001

### Comparison between different inflammation-based prognostic scores

Using OS as an endpoint, the optimal cutoff values of the different inflammation-based prognostic scores were determined using the AUC method: PNI, 42 (AUC 0.613); GPS, 1 (AUC 0.580); CAR, 0.10 (AUC 0.613); NLR, 2.55 (AUC 0.520); and PLR, 120 (AUC 0.618). The AUC values of the PNI, CAR, and PLR were the highest among the inflammation-based prognostic scores. Although the AUC value of the PNI was not the highest, the PNI was the only independent prognostic factor among the inflammation-based prognostic scores. These findings suggest that, compared to the other inflammation-based prognostic scores, the PNI is a superior prognostic factor.

### Combined prediction model

**Fig. 2:**
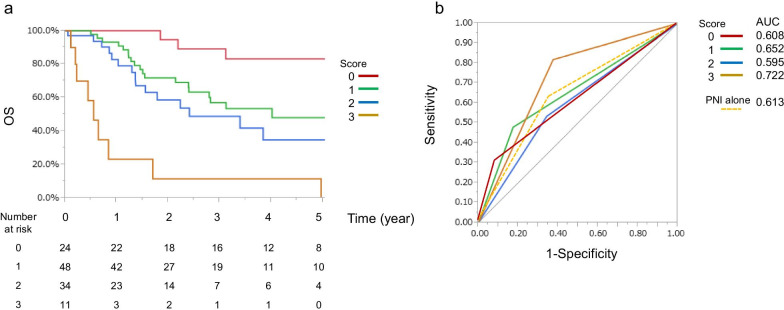
Combined prediction model. **a** Kaplan–Meier curves of OS according to the following scoring system, with 1 point assigned to each independent prognostic factor (a low PNI [< 42], lymph node metastasis, and tumor differentiation [poor]) using similar odds ratios as those reported in the multivariate analysis. The total score in the combined prediction model was calculated as the sum of the scores assigned to each independent prognostic factor. Accordingly, patients were divided into four groups, according to the number of risk factors (0, 1, 2, and 3). The proportion of patients in each group who survived was significant (*P* < 0.001). **b** Receiver operating characteristic curves of the scoring system. Predictive power was compared using the AUC for each point in the scoring system. *AUC* area under the curve, *OS* overall survival, *PNI* prognostic nutritional index

A simple scoring system was developed for all patients, with 1 point assigned to each independent prognostic factor (a low PNI [< 42], lymph node metastasis, and tumor differentiation [poor]) using similar odds ratios as those reported in the multivariate analysis. The total score in the combined prediction model was calculated as the sum of the scores assigned to each independent prognostic factor. Accordingly, patients were divided into four groups, according to the number of risk factors (0, 1, 2, and 3). The proportion of patients in each group who survived was significant (*P* < 0.001; Fig. 2a). Predictive power was compared using the AUC values for each point in the scoring system (0 [AUC 0.608], 1 [AUC 0.652], 2 [AUC 0.595], and 3 [AUC 0.722]). The AUC values for 1 and 3 points in the scoring system were higher than the AUC value for the PNI alone (AUC 0.613; Fig. 2b). A combined score of the PNI and pathological findings outperformed each marker alone, in terms of discriminatory power.

## Discussion

In this study, we showed that the PNI is associated with poor prognosis after surgical resection in patients with BTC, consistent with a previous report [7]. Tumor-related factors, including lymph node metastasis and tumor differentiation, were also found to be independent prognostic factors in multivariate analysis. Based on these findings, we developed a novel inflammation-based prognostic scoring system combining the PNI and pathological findings, which proved to be more effective than either marker alone.

A meta-analysis [22] showed that the PNI could serve as an independent prognostic factor in patients with BTC. Moreover, a high NLR and PLR may be unfavorable prognostic factors for clinical outcomes in patients with BTC [23].

The PNI, which is calculated using serum albumin concentration and total lymphocyte count, reflects the nutritional and immunological status of patients with cancer, and is a potential prognostic factor for survival. The mechanisms underlying the prognostic significance of the PNI in patients with BTC are discussed below.

Systemic inflammation has been shown to play an important role in cancer growth, invasion, and metastasis [24]. Total lymphocyte count is a component of the PNI. CD4 + and CD8 + T lymphocytes are major components of the immune microenvironment [25]. Tumor-infiltrating CD4 + and CD8 + T lymphocytes induce apoptosis and inhibit cancer cell proliferation [26]. Hence, lymphocytes play a critical role in cell-mediated antitumor immunity and immune surveillance [27]. Low lymphocyte counts lead to an insufficient immunological response in the tumor microenvironment, promoting cancer progression.

Malnutrition is common in patients with cancer [28], and has a negative impact on survival and recovery. Serum albumin concentration in the PNI reflects the nutritional status of patients with cancer. A low serum albumin concentration is associated with malnutrition and weight loss [29]. Hypoalbuminemia is not only a syndrome of poor nutritional status, but is also associated with a weakened host immune system [30]. Thus, a low serum albumin concentration usually predicts poor prognosis in patients with cancer. A low PNI may be predictive of an unfavorable prognosis in patients with BTC due to the aforementioned reason.

As discussed above, a low PNI may reflect systemic inflammation and progressive nutritional decline, resulting in poor survival. Perioperative nutritional support is recommended to improve the nutritional status of patients with hepatobiliary pancreatic carcinoma who have a high prevalence of malnutrition [31]. Preoperative immunonutrition has been reported to suppress the perioperative inflammatory response [32]. To improve prognosis, patients with a low PNI should be given immunonutrition. Further studies evaluating the relationship between immunonutrition and this inflammation-based prognostic score are required to improve the management of patients with BTC with a low PNI.

It is well known that clinicopathological characteristics, such as lymph node metastasis and tumor differentiation, significantly affect the prognosis of patients with cancer. Independent prognostic factors in this study included lymph node metastasis and tumor differentiation. Previous studies [33, 34] have shown that tumor differentiation is a predictor of survival after curative resection of BTC. In this study, patients with well-differentiated tumors had significantly longer survival times than those with other histologies. This was further confirmed by multivariate analysis. These findings suggest that tumor differentiation is a predictor of long-term survival. Patients with poorly differentiated tumors should be carefully monitored during postoperative follow-up to detect recurrence early.

Clinicopathological predictors have proven to be suboptimal for identifying high-risk patients. Recent evidence has underscored the discriminatory power of a combined prognostic index. Pinato et al*.* [35] proposed a new prognostic score for hepatocellular carcinoma, based on a combination of the modified GPS and the Cancer of the Liver Italian Program score. They reported that the predictive accuracy of the combined score was superior to that of the Cancer of the Liver Italian Program score alone. Lin et al*.* [36] combined the lymphocyte-to-monocyte ratio and pathological TNM stage to establish the inflammation-based pathological stage. They showed that the inflammation-based pathological stage was superior to either the pathological TNM stage or inflammation-based index alone. There are few established staging systems for BTC. In this study, we showed that the discriminatory power of a combined scoring system may be more effective than that of the PNI alone. Our combined scoring system accurately predicted prognosis, and may serve as a novel prognostic factor for patients with BTC. The combined scores reflected a poor prognosis, suggesting that more intensive follow-up or prophylactic postoperative treatment, such as chemotherapy and radiotherapy, is needed for patients with high scores.

The PNI was associated with several clinicopathological factors in this study. A low PNI was associated with lymph node metastasis and a longer operation time, suggesting that patients with a low PNI are at high risk of advanced disease.

This study has several limitations related to its single-center, retrospective design and small sample size. The sample size limited the statistical power of the multivariate and subgroup analyses. The study population was heterogeneous in terms of diagnosis and type of resection. OS rates differed for each type of BTC (ICC, GBC, extrahepatic cholangiocarcinoma, etc.), although not statistically significant. Most patients underwent radical resection. However, in patients with early-stage GBC, less invasive resections, such as cholecystectomy and liver bed resection, were more commonly performed. Future prospective, multicenter studies with larger sample sizes are needed to validate our findings.

## Conclusions

A high PNI, lymph node metastasis, and tumor differentiation were independent prognostic factors for OS after surgical resection in patients with BTC. Our simple and convenient scoring system will help refine patient stratification and predict survival. In addition, a novel and powerful inflammation-based scoring system was developed. Determining indications for nutritional support with immunonutrition and more intensive follow-up or postoperative treatment, such as chemotherapy and radiotherapy, is needed for patients with a high PNI.

## Data Availability

The datasets used and/or analyzed during the current study are available from the corresponding author on reasonable request.

## Abbreviations

AUC: Area under the receiver operating characteristic curve
BTC: Biliary tract cancer
CAR: CRP to albumin ratio
CRP: C-reactive protein
DFS: Disease-free survival
GBC: Gallbladder carcinoma
GPS: Glasgow Prognostic Score
ICC: Intrahepatic cholangiocarcinoma
NLR: Neutrophil to lymphocyte ratio
OS: Overall survival
PLR: Platelet to lymphocyte ratio
PNI: Prognostic nutritional index
TNM: Tumor–node–metastasis
